# Examining social-cognitive theory constructs as mediators of behaviour change in the active team smartphone physical activity program: a mediation analysis

**DOI:** 10.1186/s12889-020-10100-0

**Published:** 2021-01-07

**Authors:** Amelia V. Romeo, Sarah M. Edney, Ronald C. Plotnikoff, Tim Olds, Corneel Vandelanotte, Jillian Ryan, Rachel Curtis, Carol A. Maher

**Affiliations:** 1 School of Health Sciences, City East Campus, University of South Australia, GPO Box 2471, Adelaide, SA 5001 Australia; 2 Priority Research Centre for Physical Activity and Nutrition, University of Newcastle, University Drive, Callaghan, NSW 2308 Australia; 3 Appleton Institute, CQUniversity, Building 7, Bruce Highway, North Rockhampton, Qld 4702 Australia; 4 Commonwealth Scientific and Industrial Research Organisation, Adelaide, Australia

**Keywords:** Social-cognitive theory, Physical activity, Smartphone, App, Intervention, Mediation analysis, Mediators, Self-efficacy, Health behaviour, Behaviour change

## Abstract

**Background:**

Regular engagement in physical activity has well-established physical and psychological health benefits. Despite this, over a quarter of the global adult population is insufficiently physically active. Physical activity interventions grounded in behaviour change theory, such as the social-cognitive theory, are widely considered to be more effective than non-theoretical approaches. Such interventions set out to intervene on the ultimate outcome (physical activity), but also influence intermediate factors (social-cognitive theory constructs) which in turn, are believed to influence physical activity behaviour. The primary aim of the study was to use mediation analysis to examine whether changes in the social-cognitive theory and related constructs, in particular self-efficacy, outcome expectations, intentions, barriers and goal setting, mediated the effects of a smartphone-based social networking physical activity intervention.

**Methods:**

Mediation analyses were conducted using the PROCESS Macro in SPSS to (i) calculate the regression coefficients for the effect of the independent variable (group allocation) on the hypothesised mediators (social-cognitive theory constructs), (ii) calculate the regression coefficient for the effect of the hypothesised mediators (social-cognitive theory constructs) on the dependent variable (objectively measured physical activity or self-report physical activity), independent of group assignment and (iii) determine the total, direct and indirect intervention effects.

**Results:**

Data from 243 participants were included in the mediation analysis. There was no evidence of mediation for change in objectively measured MVPA or self-reported MVPA.

**Conclusions:**

There was no conclusive evidence that any of the social-cognitive theory constructs mediated the relationship between an app-based intervention and change in physical activity. Ongoing efforts to develop and understand components that make physical activity app-based interventions effective are recommended.

**Trial registration:**

This trial was registered with the Australian and New Zealand Clinical Trial Registry (ACTRN12617000113358, date of registration 23 January, 2017).

**Supplementary Information:**

The online version contains supplementary material available at 10.1186/s12889-020-10100-0.

## Background

Physical activity has well established psychological and physical health benefits. Most notably, engagement in regular physical activity is linked to improved muscular and cardiorespiratory fitness, functional health and mental health [1–3], as well as the prevention of primary and secondary chronic disease and decreasing all causes of mortality [4]. Despite this, over a quarter (27.5%) of the global adult population is insufficiently physically active [5]. At present, global recommendations on physical activity for health recommend adults aged between 18 and 64 years partake in at least 75 min of vigorous-intensity physical activity, or 150 min of moderate-intensity physical activity, per week [6].

Smartphones are a prominent feature in modern daily life and are developing as a promising platform for delivering public health programs. In 2017, approximately two thirds of the global adult population owned a smartphone [7] with more time being spent on a smartphone than any other device [8]. The high usage, convenience and appeal of smartphone platforms make them an attractive tool for physical activity interventions [9].

Physical activity interventions grounded in behaviour change theory, such as the social-cognitive theory, are widely considered to be more effective than non-theoretical approaches [10–12]. Such interventions typically set out to intervene on the ultimate outcome (physical activity), but also influence intermediate constructs which, in turn, are believed to influence physical activity behaviour [13]. The social-cognitive theory asserts that positively impacting the intermediate constructs of self-efficacy (the belief that an individual can effectively control their health habits) [14], outcome expectations (the expected benefits and efforts of adjusting health behaviours) [14], intentions to engage in physical activity, perceived barriers of engagement in physical activity and the setting of physical activity goals, is essential to underpin change in the target health behaviour. Basing a physical activity program on the social-cognitive theory is typically done through the inclusion of mechanisms such as social interaction and support, goal setting, feedback in real-time, rewards and incentives. In order to refine and improve interventions, it is essential to explore the effectiveness of particular components and mechanisms which produce behavioural change [15–17].

**Fig. 1:**
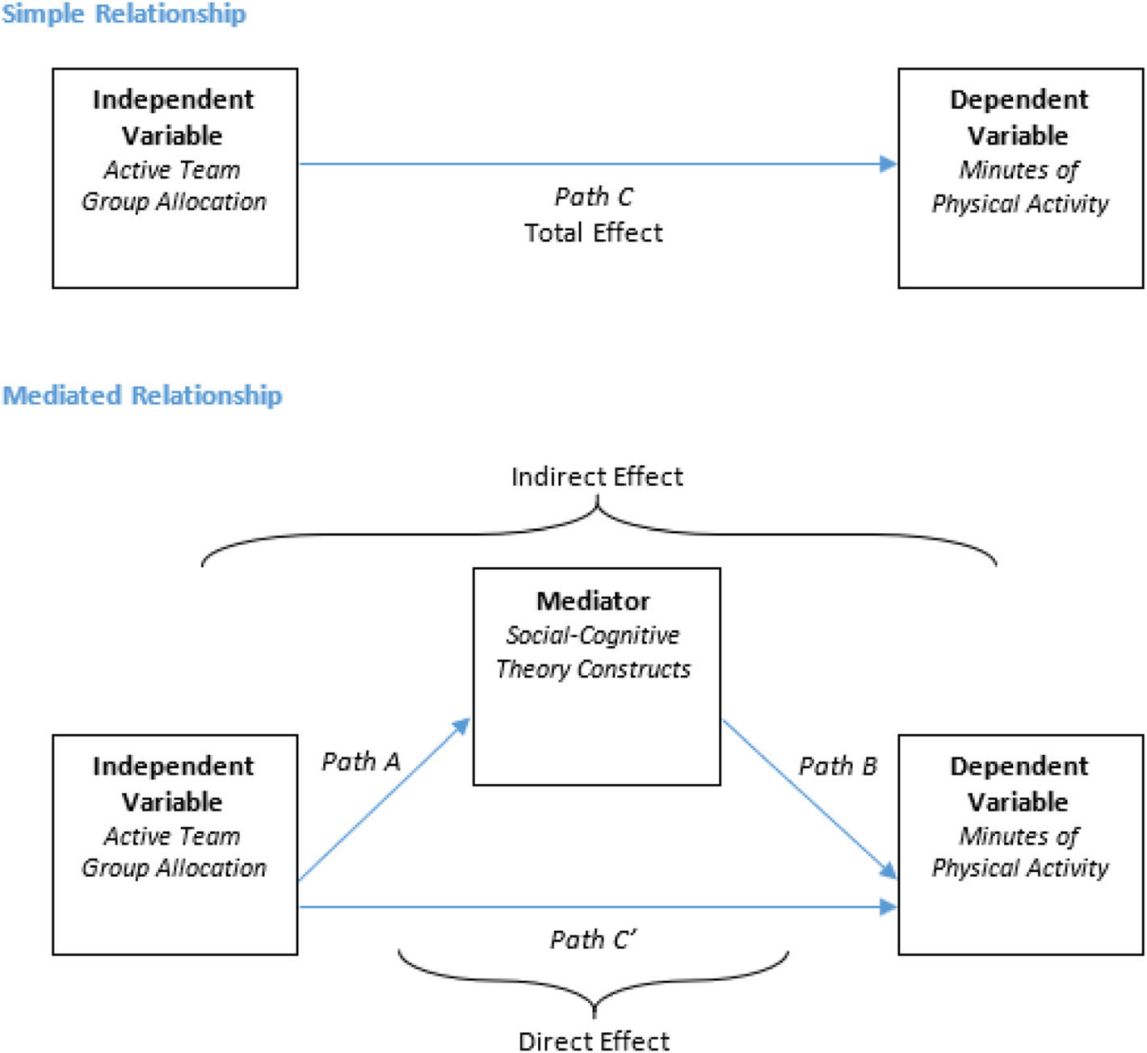
Simple and Mediated Relationship Model

One way to explore how the mechanisms of behavioural change may influence an intended outcome is through conducting a mediation analysis. A mediation analysis is a statistical approach that seeks to identify and explain factors that underlie an observed relationship between an independent variable and a dependent variable, by including a third variable known as a mediator [18, 19] (See Fig. 1 in Methods). In mediation analyses, it is assumed that mediation has occurred when the indirect effect (Path AB) is significant [20].

A handful of previous studies have examined whether the social-cognitive theory mediates the physical activity intervention’s effectiveness, with mixed findings [21]. Some studies have reported self-efficacy [22–24] and goal setting [23, 25] to have significant mediating effects on physical activity. However, others have failed to find evidence of mediation [26, 27]. The extent to which theory is embedded within intervention design varies [28], as does the mechanisms used [29]. There is some evidence to suggest that the effectiveness of theory based interventions may vary in certain subgroups, for example, based on age or sex [21]. This has been attributed to certain subgroups showing a higher degree of intrinsic motivation towards behaviour change [21].

The social-cognitive theory and related constructs have also been used to inform interventions targeting other health behaviours (i.e. weight loss or nutrition) where physical activity is a secondary outcome. Similarly, results on the effectiveness of these constructs mediating physical activity as a secondary outcome are varied [30].

Whilst a vast number of smartphone physical activity applications exist, and many contain theory-based features [31], few have been rigorously evaluated [32] and even fewer have undergone detailed analyses to understand the behaviour change mechanisms that are contributing to effective or ineffective outcomes [33]. To our knowledge, no studies to date have examined potential mediators of behaviour change in a physical activity smartphone intervention.

This study aimed to address this gap. The primary aim of this study was to examine whether changes in the social-cognitive theory and related constructs, in particular self-efficacy, outcome expectations, intentions, barriers and goal setting, mediated the effect of a smartphone-based social networking physical activity intervention. The secondary aim was to undertake hypothesis-generating analyses to determine if mediation was present within subgroups of participants based on age.

## Methods

Ethics approval was provided by the University of South Australia Human Research Ethics Committee, protocol number 0000033967. All participants provided informed consent prior to participation.

### Study design and sample size

This study is a secondary analysis using data from a large-scale randomised controlled trial (RCT) evaluating the effects of an online social networking physical activity intervention delivered via smartphone app, “Active Team”. Full details of the RCT are provided elsewhere [34]. Briefly, four-hundred and forty-four (444) participants were recruited between October 2016 to December 2017. Participants were aged 18–65 years old, Australian residents, fluent in English, self-reported attaining less than 150 min of moderate-to-vigorous physical activity (MVPA) per week at time of enrolment, and used Facebook. Participants enrolled in the study in clusters of three to eight Facebook friends, the first participant joining the intervention was designated as the team “captain” and their friends as their “team members”. Participant clusters were randomly allocated to one of three groups: a socially-enhanced intervention group, basic intervention group and waitlist control group. This mediation analysis is based on data from the socially-enhanced intervention group (*n*= 141) and the waitlist control group (*n*= 143). The basic intervention group was excluded from this analysis due to its features not being based on the social-cognitive theory.

### Intervention details

Participant clusters allocated to the socially-enhanced intervention group received a pedometer, access to the Active Team app and were challenged with the goal of taking 10,000 steps a day for 100 days. The Active Team app features were developing based on the social-cognitive theory [35], and incorporates individuals’ pre-existing social networks by linking to Facebook and encouraging social interaction and enjoyment through social and gamified features such as ability to send virtual gifts, compete in mini challenges, view progress on a leader board, unlock features, and post messages and photographs to the Facebook-style newsfeed. Participant clusters allocated to the waitlist control group were instructed to go about their usual daily activities and received access to the Active Team program at the end of the study. Full details of the study’s main findings are published elsewhere [36]. Briefly, from baseline to post-intervention (3-month assessment), objectively measured MVPA increased by an average of 11 (SD 329) minutes/week in the socially-enhanced intervention group and increased by an average of 3 (SD 316) minutes/week in the waitlist control group (non-significant difference between groups). Over the same period, self-reported MVPA increased by an average of 181 (SD 316) minutes/week in the socially-enhanced intervention group, compared to an average increase of 93 (SD 288) minutes/week in the waitlist control group. Subgroup analyses suggested that intervention effectiveness was associated with age (*p*=0.002) but not sex, BMI, or education.

### Outcome measures

Outcome measures were assessed at baseline, end of program (i.e., 3 months post baseline) and 6 months post program completion (i.e., 9 months post baseline). Change in MVPA from baseline to 3 months for objective and self-reported physical activity has been used for this analysis.

### Physical activity (objective)

Objectively measured MVPA was collected using wrist-worn GENEActiv accelerometers for 7-days at each assessment time point. The GENEActiv accelerometers output raw data regarding the frequency, duration and intensity of physical activity [37]. They are highly reliable, with intra-instrument and inter-instrument coefficients of variation of 1.8 and 2.4% respectively [38]. Similarly, when compared in a mechanical shaker, GENEActiv accelerometers have excellent validity (r=0.89) [38]. Minimum wear criteria were used to determine valid accelerometer data for inclusion in data analyses: participants must have worn the accelerometer for at least 10 waking hours, on four or more days, including one weekend day. Participants returning incomplete data were asked to wear the GENEActiv accelerometer again up to two more times [36]. Accelerometer files were processed with 60 s epochs and the Esliger cutpoints [38] were used to define MVPA as any activity above 645 counts per minute. Daily average (weighted as 5X [weekday average] + 2X [weekend day average]/7) was multiplied by seven to calculate mean weekly minutes of objective MVPA.

### Physical activity (self-reported)

Self-reported MVPA data were collected using the 8-item Active Australia Survey (AAS). The AAS asks participants to recall the frequency, duration, intensity and type of physical activity performed within the previous week [39]. For example, “In the last week, how many times have you walked continuously for at least 10 minutes, for recreation, exercise or to get to or from places?” and “What do you estimate was the total time you spent walking in this way in the last week?”*.* The AAS has acceptable repeatability when compared with three other self-reported physical activity measures (*k=* 0.52 95%CI 0.44–0.60) [40] and validity compared with accelerometery data (r_s_ = 0.61 95%CI 0.43–0.75) [41].

### Social cognitive theory measures

The constructs self-efficacy, outcome expectations, intentions, perceived barriers, and goals in relation to participating in regular physical activity were assessed at each time point via 21-items.

Self-efficacy for physical activity under varying circumstances (i.e., tired, in a bad mood, do it alone, when it becomes boring, can’t notice fitness improvements, competing demands on time, feel stiff or sore, bad weather) was assessed via 8 items (Cronbach’s α = 0.90) [42] and responses were on a 5-point Likert scale ranging from “not at all confident” to “extremely confident” [42].

Outcome expectations related to potential positive benefits of physical activity (i.e., reduce tension or manage stress, confidence about health, better sleep, positive outlook, control weight) were assessed via 5 items (Cronbach’s α = 0.83) [42]. Participants responded on a 5-point Likert scale ranging from “strongly disagree” to “strongly agree” [42].

Intentions were assessed via 2 items (i.e., motivated, determined) to engage in regular physical activity (Cronbach’s α = 0.91) [43]. Participants responded on a 5-point Likert scale ranging from “not at all motivated/determined” to “extremely motivated/determined” [43].

Perceived barriers to participating in physical activity were assessed using 5 items (i.e., take too much time, less time with friends and family, too many other responsibilities, worry about looking awkward, would cost too much money, Cronbach’s α = 0.72) [42], responses were on a 5-point Likert scale ranging from “strongly disagree” to “strongly agree” [42].

Goals relative to physical activity was assessed with a single-item (i.e., “I often set physical activity goals”) with a 5-point Likert scale response option ranging from “strongly disagree” to “strongly agree” [44].

Sociodemographic variables (e.g., age, sex, education) were also collected at baseline. These variables were self-reported and included age, sex (male or female), education level (high school or less; technical or further education; university degree or higher), height and weight from which body mass index (BMI) was calculated and marital status (single, partner, prefer not to say).

### Statistical analyses

Change in MVPA from baseline to 3 months was calculated for objective and self-reported physical activity. Outliers were detected on the basis of change scores falling outside of 3.0 standard deviations from the mean [45], resulting in three outliers being removed from the self-reported MVPA data (lower limit − 843 min/week, upper limit 1249 min/week).

Simple mediation models were conducted with the intervention allocation (either control or socially-enhanced group) as the independent variable. Mediator variables were mean difference from baseline to 3 months in the social-cognitive theory constructs of self-efficacy, outcome expectations, intentions, barriers and goals, with each construct tested individually. The dependent variables were mean differences from baseline to 3 months in objective and self-reported MVPA. Mediation analyses were conducted in IBM SPSS Statistics, Version 23 using the PROCESS INDIRECT Macro (Model 4) [19] to (i) calculate the unstandardized regression coefficients for the effect of the independent variable (intervention allocation) on the hypothesised mediators (social-cognitive theory construct change scores) (Path A), (ii) calculate the unstandardized regression coefficient for the effect of the hypothesised mediators (social-cognitive theory construct change scores) on the dependent variable (physical activity change scores), independent of intervention allocation (Path B) and (iii) determine the total (Path C), direct (Path C′) and indirect (Path AB) intervention effects (see Fig. 1).

The PROCESS macro automatically handles missing data through listwise deletion, which was considered appropriate as Little’s test accepted the null-hypothesis that the data were missing completely at random (*X*^2^= 4.877 df = 7, *P* = .675). As such, only participants with complete data were included in the mediation analysis (243 participants when the dependent variable was self-reported MVPA and 222 participants when the dependent variable was objectively measured MVPA).

The PROCESS macro generates bias-corrected bootstrapped 95% asymmetrical confidence intervals around the indirect effect. For mediation to be present, the indirect effect (Path AB) must be significant [19, 20]. Using Rucker and colleagues’ [20] recommendations for mediation analyses, mediation will be explored even in the absence of a significant total (Path C) or direct (Path C′) effect.

Given that intervention effectiveness was associated with age, but not other sociodemographic variables (sex, BMI, education category) (see Additional File 1), a subgroup analysis was conducted to determine whether social-cognitive theory variables mediated intervention effectiveness in older and younger participants. Participants were dichotomised based on being ≤40 years of age and > 40 years of age. Treating age in this manner meant that if findings were significant (i.e. that social-cognitive theory variables mediated intervention effects in one age group but not the other) their relevance for future research would be clear to interpret (i.e. suggesting that future social-cognitive theory physical activity interventions may be more fruitful if they were targeted at older or younger adults). For all analyses, an alpha < 0.05 was used to denote statistical significance, with Holm’s Sequential Bonferroni adjustment applied to reduce the risk of Type 1 errors (false positives) due to multiple comparisons. All analyses were conducted in IBM SPSS Statistics, Version 23.

## Results

Table 1 presents baseline characteristics of the study participants. The mean age of participants was 41.5 (SD 11.3) years, the majority (74.8%) were female. The mean BMI of participants was 30.0 (SD 6.8) which is categorised as on the border between overweight and obese [46]. Baseline, 3-month and mean change values for the mediator variables and dependent variables are detailed in Table 2.

**Table 1:** Baseline characteristics of study participants

Characteristic	Split group characteristics		Total
	Intervention (*n*= 129)	Control (*n*= 129)	(*n*=258)
	mean (SD)	mean (SD)	mean (SD)
Age (years)	43.45 (11.39)	39.61 (10.86)	41.53 (11.27)
Sex Female (%)	75.20	74.40	74.80
Married/Partner (%)	77.50	78.30	77.90
Completed Post-School Qualifications (%)	82.90	86.00	84.50
BMI^a^	30.80 (6.94)	29.10 (6.51)	29.95 (6.77)
Self-reported MVPA (weekly)	243.71 (199.66)	270.67 (277.04)	257.14 (241.22)
GeneActiv MVPA (weekly)	742.84 (373.8)	759.43 (348.32)	751.17 (360.64)

^a^ Note that BMI is calculated from self-reported height and weight

**Table 2:** Baseline, 3-month and mean change in mediator and dependent variables for intervention and control groups

Characteristic	Split group characteristics					
	Intervention (*n*= 122)			Control (*n*= 121)		
	Baseline mean (SD)	3-month mean (SD)	Change Mean (SD)	Baseline mean (SD)	3-month mean (SD)	Change Mean (SD)
Self-Efficacy	2.96 (0.71)	2.81 (0.82)	−0.15 (0.81)	2.95 (0.75)	2.70 (0.76)	− 0.25 (0.77)
Outcome Expectations	4.32 (0.57)	3.94 (0.39)	−0.38 (0.58)	4.36 (0.53)	4.17 (0.52)	−0.19 (0.47)
Intentions	4.09 (0.59)	3.78 (0.88)	−0.31 (0.85)	3.94 (0.64)	3.64 (0.79)	−0.31 (0.78)
Perceived Barriers	2.20 (0.56)	2.29 (0.58)	0.09 (0.52)	2.38 (0.74)	2.50 (0.61)	0.12 (0.73)
Goals	3.10 (1.02)	3.30 (1.01)	0.20 (1.03)	2.98 (0.98)	3.00 (0.99)	0.02 (1.01)
Self-reported MVPA^a^ (weekly)	240.15 (189.18)	421.25 (323.17)	181.10 (316.19)	272.45 (282.17)	365.45 (308.93)	92.99 (288.26)
GeneActiv MVPA^a^ (weekly)	735.56 (374.22)	746.41 (365.47)	10.71 (329.21)	758.03 (354.97)	760.76 (371.91)	2.73 (315.91)

### Mediation analysis

Data for all pathways within the simple and mediated relationship model has been reported in Table 3 and Table 4. All reported values in text and in tables are unstandardized regression coefficients, adjusted for baseline values. The indirect effect of the intervention on objective MVPA and self-reported MVPA data was examined to determine the presence of mediation. There was no statistically significant indirect effect when any of the potentially mediating variables; self-efficacy, intentions, outcome expectations, perceived barriers and goals were included in the model for objective MVPA (see Table 3). Similarly, there was no statistically significant indirect effect when any of the social-cognitive theory constructs were examined as mediators of self-reported MVPA (see Table 4). As such, the criteria for mediation were not satisfied.

**Table 3:** Results of the mediation model for objectively measured MVPA. *N* = 222

Hypothesised mediator	A (SE)	B (SE)	C (SE)	C′ (SE)	AB (SE) [95% CI]
Change in self-efficacy	0.057 (0.054)	5.340 (3.918)	1.985 (3.125)	1.671 (3.127)	0.313 (0.376) [−0.23, 1.204]
Change in intentions	−0.007 (0.055)	− 0.323 (3.835)	1.985 (3.125)	1.983 (3.133)	0.002 (0.224) [−0.574, 0.481]
Change in outcome expectations	−0.135 (0.036)* ^∧^	9.803 (5.896)	1.985 (3.125)	3.312 (3.214)	−1.328 (1.178) [−4.244, 0.287]
Change in perceived barriers	−0.017 (0.044)	4.423 (4.811)	1.985 (3.125)	2.061 (3.128)	−0.076 (0.376) [− 0.980, 0.574]
Change in goals	0.109 (0.070)	0.856 (3.059)	1.985 (3.125)	1.891 (3.150)	0.093 (0.326) [−0.589, 0.768]

Note: * denotes: *P* < 0.05, ^∧^ denotes: *P* remains significant after Holm’s Bonferroni adjustment

*SE* Standard Error, *CI* Confidence Interval

*MVPA* moderate-to-vigorous physical activity

A = intervention allocation effect on mediators

B = association between mediators and change in objectively measured MVPA

C = total effect model

C′ = direct effect of intervention allocation on change in objectively measured MVPA

AB = indirect effect of intervention allocation on change in objectively measured MVPA through the hypothesised mediator

**Table 4:** Results of the mediation model for self-reported MVPA. *N*=243

Hypothesised mediator	A (SE)	B (SE)	C (SE)	C′ (SE)	AB (SE) [95% CI]
Change in self-efficacy	0.046 (0.050)	39.146 (24.690)	44.053 (19.412)* ^∧^	42.269 (19.384)* ^∧^	1.784 (2.539) [−2.194, 8.066]
Change in intentions	− 0.003 (0.052)	71.507 (23.613)* ^∧^	44.053 (19.412)* ^∧^	44.257 (19.092)*	− 0.203 (3.924) [−9.314, 6.588]
Change in outcome expectations	− 0.142 (0.034)* ^∧^	5.171 (36.696)	44.053 (19.412)* ^∧^	44.789 (20.140)* ^∧^	− 0.735 (7.581) [− 18.656, 11.204]
Change in perceived barriers	− 0.017 (0.041)	− 51.389 (30.509)	44.053 (19.412)* ^∧^	43.186 (19.346)*	0.868 (2.388) [− 3.494, 6.396]
Change in goals	0.901 (0.065)	32.794 (19.090)	44.053 (19.412)* ^∧^	41.099 (19.411)* ^∧^	2.955 (2.853) [−2.134, 8.995]

Note: * denotes: *P* < 0.05, ^∧^ denotes: *P* remains significant after Holm’s Bonferroni adjustment

*SE* Standard Error, *CI* Confidence Interval

*MVPA* moderate-to-vigorous physical activity

A = intervention allocation effect on mediators

B = association between mediators and change in self-reported MVPA

C = total effect model

C′ = direct effect of intervention allocation on change in self-reported MVPA

AB = indirect effect of intervention allocation on change in self-reported MVPA through the hypothesised mediator

### Subgroup analysis

A mediation analysis was undertaken on the basis of age subgroups (younger adults [18–40 years; Table 5] and older adults [> 40 years; Table 6]). Amongst participants aged 18–40 years, no statistically significant indirect effect was identified when any of the social-cognitive theory constructs were the mediator variable. Thus, mediation was not present for any of the social-cognitive theory constructs within this sub-group. Similarly, there was no statistically significant indirect effect when any of the social-cognitive theory constructs were the mediator variable in the sub-group of participants aged 41 years or older, again, indicating no mediation.

**Table 5:** Results of the mediation model for participants aged 18–40 years. *N*=124

Hypothesised mediator	A (SE)	B (SE)	C (SE)	C′ (SE)	AB (SE) [95% CI]
Change in self-efficacy	−0.041 (0.027)	16.792 (36.586)	−10.940 (29.130)	−10.250 (29.263)	−0.690 (2.617) [− 7.275, 4.623]
Change in intentions	−0.032 (0.077)	3.292 (34.677)	−10.940 (29.130)	− 10.834 (29.700)	− 0.106 (3.559) [− 10.575, 4.676]
Change in outcome expectations	− 0.116 (0.044)*	49.986 (59.629)	−10.940 (29.130)	−5.156 (29.971)	−5.784 (8.482) [− 25.544, 8.578]
Change in perceived barriers	0.017 (0.061)	− 23.031 (43.379)	−10.940 (29.130)	− 10.548 (29.225)	−0.392 (2.686) [−6.873, 4.990]
Change in goals	0.103 (0.089)	26.496 (29.684)	−10.940 (29.130)	−13.681 (29.315)	2.741 (4.010) [−5.504, 11.537]

Note: * denotes: *P* < 0.05, ^∧^ denotes: *P* remains significant after Holm’s Bonferroni adjustment

*SE* Standard Error, *CI* Confidence Interval

A = intervention allocation effect on mediators

B = association between mediators and change in self-reported MVPA

C = total effect model

C′ = direct effect of intervention allocation on change in self-reported MVPA

AB = indirect effect of intervention allocation on change in self-reported MVPA through the hypothesised mediator

**Table 6:** Results of the mediation model for participants aged 41 years or older. *N*=122

Hypothesised mediator	A (SE)	B (SE)	C (SE)	C′ (SE)	AB (SE) [95% CI]
Change in self-efficacy	0.103 (0.069)	27.000 (38.290)	82.665 (28.993)*	79.889 (29.320)* ^∧^	2.776 (4.036) [−3.891, 12.899]
Change in intentions	0.144 (0.071)	118.663 (35.714)* ^∧^	82.665 (28.993)*	80.954 (27.856)*	1.712 (8.429) [−15.237, 18.537]
Change in outcome expectations	− 0.019 (0.052)* ^∧^	− 43.151 (60.930)	82.665 (28.993)*	74.547 (30.568)*	8.118 (14.734) [− 26.831, 30.787]
Change in perceived barriers	− 0.026 (0.055)	− 88.453 (47.934)	82.665 (28.993)*	80.352 (28.734)* ^∧^	2.313 (5.786) [− 7.333, 16.644]
Change in goals	0.061 (0.096)	40.684 (27.521)	82.665 (28.993)* ^∧^	80.104 (28.900)* ^∧^	2.481 (5.004) [− 7.451, 13.440]

Note: * denotes: *P* < 0.05, ^∧^ denotes: *P* remains significant after Holm’s Bonferroni adjustment

*SE* Standard Error, *CI* Confidence Interval

A = intervention allocation effect on mediators

B = association between mediators and change in self-reported MVPA

C = total effect model

C′ = direct effect of intervention allocation on change in self-reported MVPA

AB = indirect effect of intervention allocation on change in self-reported MVPA through the hypothesised mediator

## Discussion

The purpose of this study was to determine if the social-cognitive theory and related constructs of self-efficacy, outcome expectations, intentions, perceived barriers and goals; mediated change in physical activity for participants of an app-based physical activity intervention. For both objective MVPA and self-reported MVPA, the indirect effects were not significant and as such there was no evidence of mediation. Furthermore, there was no conclusive evidence of mediation within subgroups of participants based on age. Thus, in all, this study failed to find evidence of mediation for any of the social-cognitive theory constructs.

The lack of evidence supporting the notion that social-cognitive theory constructs mediate the effects of a physical activity intervention is consistent with previous studies which similarly reported no evidence of mediation [26, 27, 47]. Only a small change from baseline to 3-months was recorded in all social-cognitive theory constructs within this study. Notably, this change was in a negative (i.e. undesirable) direction for the constructs of self-efficacy, outcome expectations and intentions. Whilst on the face of it, this result is surprising, it is actually consistent with results from previous mediation analyses of physical activity interventions that have also reported a negative direction of change for self-efficacy [48, 49] and outcome expectancy [48] from baseline to follow-up. One explanation for the negative direction of change may be initial elevation bias: participants may have overestimated their baseline function for the social-cognitive theory constructs, thus leaving minimal room for improvement [50]. This is particularly true for the constructs of outcome expectations and intentions to engage in physical activity, where mean baseline data for the intervention group was 4.32 (SD 0.57) and 4.09 (SD 0.59) out of a maximum five respectively. Initial elevation bias is common in self-reported data [50], and particularly self-reported data of internal states [50] as is the social-cognitive theory constructs.

Alternatively, the negative direction of change for the constructs of self-efficacy, outcome expectations and intentions may be explained by the response-shift theory. The response-shift theory suggests changes in a measured variable (e.g. physical activity) may lead to change in an individual’s self-evaluation [51]. For example, prior to engaging in the Active Team smartphone physical activity program, participants may have had optimistic expectations toward changing physical activity behaviour. However, once engaging in the program it is possible participants’ experiences of barriers and constraints prompted a realisation that changing physical activity behaviour is more difficult than initially perceived. Whilst the response-shift theory is yet to be explored in depth within the social-cognitive domain [52], it may provide an explanation for the small and negative direction of change in the social-cognitive theory constructs.

This study’s finding that the social-cognitive theory constructs did not appear to mediate the relationship between the intervention and change in physical activity adds to the ongoing debate about the role of theory in behaviour change programs. Conventionally, it is widely accepted that theory-based physical activity interventions are more effective than non-theoretical approaches [10–12]. However, two recent meta-analyses have questioned this, finding that interventions based on the social-cognitive theory were no more effective than non-theoretical based interventions [28, 29]. The contrasting findings suggest that the role and implementation of theory in intervention design is not well understood.

Rather than theory having no role, it is possible our intervention, and others that have failed to demonstrate mediation, may not have operationalised social-cognitive theory effectively. One systematic review reported that interventions which extensively incorporated theory into their design had larger effect sizes than those with less or no use of theory [29]. The Active Team intervention design was informed by the social-cognitive theory; however, design emphasis was on usability and enjoyability. This is in contrast to previous physical activity interventions which emphasised the social-cognitive theory through the incorporation of educational modules [22, 23, 25]. This study’s finding of no statistically-significant mediating effects of social-cognitive theory constructs on physical activity, may be a consequence of the social-cognitive theory solely guiding the development of the smartphone app intervention and not being a prominent feature of the intervention, as reported in previous studies [29, 53].

In light of these findings, the social-cognitive theory could be more wholly embedded within the Active Team intervention in a number of ways; personalised step goals, adjusted according to user performance and preference to enhance self-efficacy; use of notifications/reminders to improve users’ outcome expectations regarding physical activity (i.e. improved vitality [54], functional health [1], mental wellbeing [55] and social opportunity [56]); inclusion of season-based app features, goals and social challenges (e.g. indoor challenges during winter when the weather is likely to be poor and outdoor challenges during warmer weather months) to address perceived barriers. These additional features aligned with the social-cognitive theory components may produce a more significant change in the mediator variables [29].

A strength of this study is that it is the first to attempt to examine the mediating effect of the social-cognitive theory constructs in the context of an app-based physical activity intervention. Additional strengths include the study’s large sample size, use of established social-cognitive theory measurement tools and high-quality outcome measures, including accelerometry.

It is important to acknowledge limitations, including the use of self-reported physical activity measures, which are susceptible to social desirability bias, response bias, initial elevation bias and recall bias [57]. In addition, our analysis approach focused on change scores, which can be susceptible to measurement error and does not account for baseline values [58]. Furthermore, this mediation analysis was conducted as a secondary analysis, rather than being an experiment designed with a primary focus on understanding intervention mechanisms [59]. The PROCESS Macro in SPSS does not account for clustered data, however since this mediation analysis did not find evidence of mediation, accounting for clustering will not change the results. Additionally, the social-cognitive theory tools used in this study lack comprehensive evidence of reliability, validity and sensitivity to change. They were selected after extensive literature searching which failed to identify alternatives with established psychometric properties, thus the current tools were selected on the basis that they had been used in previous research. Given these limitations, the results of this study should be interpreted with caution.

The use of smartphone apps as a platform for delivering physical activity interventions is in its infancy. This study is the first mediation analysis of an app-based physical activity intervention. As such it is important to report all findings, including those that may be non-supportive, as they can contribute to understanding of imperative and unnecessary intervention components, to then facilitate the development of more effective intervention designs [60–62].

## Conclusion

There was no conclusive evidence that any of the social-cognitive theory constructs of self-efficacy, outcome expectations, intentions, perceived barriers or goals mediated the relationship between an app-based intervention and change in physical activity. Ongoing efforts to develop and understand components that make physical activity app-based interventions effective are recommended.

## Supplementary Information

**Additional file 1.** Correlation Matrix.

## Data Availability

The datasets used and/or analysed during the current study are available from the corresponding author on reasonable request.

## Abbreviations

AAS: Active Australia Survey
BMI: Body mass index
MVPA: Moderate to vigorous physical activity
RCT: Randomised controlled trial
