# Supplementary material for: Examining social-cognitive theory constructs as mediators of behaviour change in the active team smartphone physical activity program: a mediation analysis
Source: BMC Public Health. 2021 Jan 7;21:88. doi: 10.1186/s12889-020-10100-0 (PMC7792171; doi:10.1186/s12889-020-10100-0)
Supplement: Supplementary file 1 — Additional file 1. Correlation Matrix. [file 12889_2020_10100_MOESM1_ESM.docx]

### Additional file 1: Correlation Matrix

|  | | | Change in Self-Report PA T2-T1 | Gender | Age at start of challenge in years | BMI Category | Captain or team member? | Education Category | Marital Status |
| --- | --- | --- | --- | --- | --- | --- | --- | --- | --- |
| Spearman's rho | Change in Self-Report PA T2-T1 | Correlation Coefficient | 1.000 |  |  |  |  |  |  |
|  |  | Sig. (2-tailed) |  |  |  |  |  |  |  |
|  |  | N | 243 |  |  |  |  |  |  |
|  | GENDER | Correlation Coefficient | -0.007 | 1.000 |  |  |  |  |  |
|  |  | Sig. (2-tailed) | 0.914 |  |  |  |  |  |  |
|  |  | N | 243 | 243 |  |  |  |  |  |
|  | Age at start of challenge in years | Correlation Coefficient | .196^**^ | -0.002 | 1.000 |  |  |  |  |
|  |  | Sig. (2-tailed) | 0.002 | 0.973 |  |  |  |  |  |
|  |  | N | 243 | 243 | 243 |  |  |  |  |
|  | BMI_CAT | Correlation Coefficient | -0.045 | -0.062 | .217^**^ | 1.000 |  |  |  |
|  |  | Sig. (2-tailed) | 0.481 | 0.335 | 0.001 |  |  |  |  |
|  |  | N | 243 | 243 | 243 | 243 |  |  |  |
|  | Captain (1) or team member? (2) | Correlation Coefficient | -0.026 | -0.063 | -0.122 | -0.005 | 1.000 |  |  |
|  |  | Sig. (2-tailed) | 0.683 | 0.332 | 0.058 | 0.937 |  |  |  |
|  |  | N | 243 | 243 | 243 | 243 | 243 |  |  |
|  | EDU_CAT | Correlation Coefficient | 0.031 | .135^*^ | -0.064 | -0.066 | -0.040 | 1.000 |  |
|  |  | Sig. (2-tailed) | 0.631 | 0.036 | 0.319 | 0.305 | 0.537 |  |  |
|  |  | N | 243 | 243 | 243 | 243 | 243 | 243 |  |
|  | MARITAL_STATUS | Correlation Coefficient | -0.073 | 0.033 | -.219^**^ | -0.073 | 0.097 | -0.116 | 1.000 |
|  |  | Sig. (2-tailed) | 0.258 | 0.614 | 0.001 | 0.259 | 0.131 | 0.071 |  |
|  |  | N | 243 | 243 | 243 | 243 | 243 | 243 | 243 |
| **. Correlation is significant at the 0.01 level (2-tailed). | | | | | | | | | |
| *. Correlation is significant at the 0.05 level (2-tailed). | | | | | | | | | |
| a. Valid or missing data = valid data | | | | | | | | | |
